# Antioxidant Activity of *Paederia foetida* Linn. Leaf Extract and Its Effect on Bovine Sperm Quality

**DOI:** 10.3390/vetsci12080775

**Published:** 2025-08-19

**Authors:** Sasitorn Phankhieo, Jiraporn Laoung-on, Ranida Quiggins, Pimchanok Nuchniyom, Paiwan Sudwan

**Affiliations:** 1 Department of Anatomy, Faculty of Medicine, Chiang Mai University, Chiang Mai 50200, Thailand; sasitorn_pha@cmu.ac.th (S.P.); ranida.quiggins@cmu.ac.th (R.Q.);; 2 Office of Research Administration, Chiang Mai University, Chiang Mai 50200, Thailand; 3 Research Institute for Health Sciences (RIHES), Chiang Mai University, Chiang Mai 50200, Thailand

**Keywords:** *P. foetida*, phytochemical, flavonoid, phenolic, sperm motility, sperm morphology, sperm viability, oxidative stress

## Abstract

*Paederia foetida* Linn. (*P. foetida*) is a wild vegetable native to both temperate and tropical regions of Asia, including Thailand. Traditionally, its leaves are believed to offer various health benefits, including promoting longevity and enhancing sexual performance. The purpose of the present study is to examine the phytochemical constituents and antioxidant efficacy and determine the influence on bovine sperm quality of aqueous leaf extracts. The powder of leaves was extracted with distilled water at 80 °C and analyzed for phytochemicals using HPLC for antioxidant properties and effects on sperm. Sperm samples were divided into five groups and treated with extract concentrations of 0.1375, 0.275, 0.55, 1.10, and 2.20 mg/mL and compared with a control (Krebs solution). The extract contained phenolic compounds and flavonoids. It exhibited potent scavenging efficacy against DPPH and ABTS radicals and inhibited LPO but showed low reduction of Fe (III) to Fe (II) and low inhibition of AOPP formation. No significant changes in sperm quality were observed with 0.1375, 0.275, 0.55, and 1.10 mg/mL, while 2.20 mg/mL caused a significant increase in abnormal sperm. In conclusion, *P. foetida* leaf extract has antioxidant potential and at concentrations up to 1.10 mg/mL is not detrimental to sperm quality.

## Simple Summary

Our research investigated the indigenous plant *P. foetida* to identify phytochemical compounds and evaluate antioxidant potential and its effect on sperm quality. The findings revealed that the extract contained phytochemicals, particularly flavonoids and phenolic compounds, which exhibited antioxidant activity. At a concentration below 1.10 mg/mL, the extract did not exert toxic effects on sperm motility, viability, acrosome integrity, or morphology. However, at a higher concentration (2.20 mg/mL), the extract failed to enhance these sperm quality parameters and instead led to elevated oxidative stress. These results suggest that this traditional plant possesses both beneficial and potentially harmful effects, depending on the quantity used, and highlight its potential as a natural antioxidant to support sperm quality when used appropriately.

## 1. Introduction

Currently, male factors contribute to infertility, and such issues can be identified through semen analysis, indicating abnormalities in sperm parameters [1]. The World Health Organization (WHO) highlights that sperm analysis is crucial for diagnosing male infertility since it measures key factors like semen volume, sperm count, morphology, and motility, which together provide insight into fertility status [2,3]. Furthermore, normal sperm motility and morphology have shown a positive association with successful fertilization and pregnancy outcomes [3]. Among the primary causes of male infertility is oxidative stress, in which reactive oxygen species (ROS) induce cellular damage, which in turn promotes lipid membrane degradation and DNA fragmentation, eventually affecting sperm motility and embryonic developmental competence [4]. The ROS levels in semen are regulated by antioxidants; however, oxidative stress leads to an imbalance between ROS and these antioxidants [5,6]. Numerous factors associated with an unhealthy lifestyle, such as smoking, excessive alcohol intake, poor dietary habits, psychological stress, a sedentary lifestyle, sleep duration and sleep quality, obesity, infections, inflammatory responses, and environmental exposures (e.g., radiation, metals, and toxic pollutants), have been shown to elevate ROS levels in sperm, thus contributing to male infertility [7,8,9,10,11]. Overwhelming oxidative stress from these factors can adversely affect both the male reproductive system and sperm count, motility, and morphology of the semen, resulting in a decrease in overall semen quality [12,13]. Various scientific studies have demonstrated the therapeutic benefits of medicinal plants. For instance, *Nelumbo nucifera* petals and *Zingiber officinale* have shown remedial effects against oxidative stress in male reproductive function and have neuro- and hepatoprotective properties in a mancozeb-induced rat model [14,15,16,17,18,19]. Additionally, *Boesenbergia rotunda* rhizomes have been shown to enhance the seminiferous tubules, testis, and seminal vesicle [20]. *Kaempferia parviflora* rhizomes may promote spermatogenesis by stimulating Sertoli cell activity, thereby increasing both sperm density and secretory granules [21]. *Moringa oleifera* leaf extracts help promote sexual health and benefit the male reproductive system [22]. Furthermore, the stamens of *Bombax ceiba* have demonstrated the ability to scavenge free radicals and increase antioxidant activity, leading to increased sperm motility, sperm viability, and normal sperm morphology [23].

*Paederia foetida* Linn. (*P. foetida*) belongs to the Rubiaceae family and is alternatively named *Paederia scandan*, with foetida being a Latin word meaning “foul-smelling” or “stinky” [24,25]. This plant is indigenous to both temperate and tropical regions of Asia, extending from Japan, India, and Southeast Asia [26,27,28]. *P. foetida* can grow across a range of habitats, from submerged wetlands to arid sandhill environments to desolate wastelands; however, it favors sunny floodplains and low-lying areas [27]. The plant is characterized by a bitter taste and the a pungent emission of a sulfurous scent when its leaves or steams are crushed or bruised [25,27,29].

*P. foetida*, traditionally used as both food and medicine [30], provides some protein and carbohydrates while being exceptionally high in vitamin C and essential minerals (calcium, potassium, and iron) [31]. Beyond its nutritional value, traditional folk medicine has utilized *P. foetida* as a treatment for different diseases, including dysentery, diarrhea, piles, gout, renal calculi, emesis, gastric ulcers, and different types of inflammation [32,33,34]. It has also been reported to have antidiarrheal [35], antihyperlipidemic, antihyperglycemic [32], antinociceptive [34], anti-inflammatory [36], antibacterial [37], antidiabetic [28,38], and antioxidant activity [29,39]. Moreover, in a previous study, *P. foetida* was shown to increase sexual behaviors in rats [40]. The therapeutic efficacy of the plant is due to its phytochemical compounds, which exert specific biological effects on the human body [29]. Previous studies reported that *P. foetida* has strong antioxidant activity, with dried leaves exhibiting higher antioxidant potential than fresh leaves [41]. This plant has been shown to contain phenolic compounds, alkaloids, tannins, caffeic acid, quercetin, flavonoids, glycosides, and kaempferol [27,42,43,44,45]. These compounds are capable of increasing male reproductive ability [14,15,46].

Traditional beliefs in certain regions of Thailand suggest that daily consumption of *P. foetida* leaves may act as an elixir of longevity and enhance sexual performance. Nevertheless, the reproductive effects of *P. foetida* remain under-researched, and these folkloric claims lack sufficient scientific and medical validation. Therefore, this study focuses on examining the phytochemical constituents in *P. foetida* as an herbal tea preparation, its antioxidant activity, and its impact on reproductive function, utilizing sperm cells as an in vitro biological model.

## 2. Materials and Methods

### 2.1. Plant Collection and Extraction

**Figure 1:**
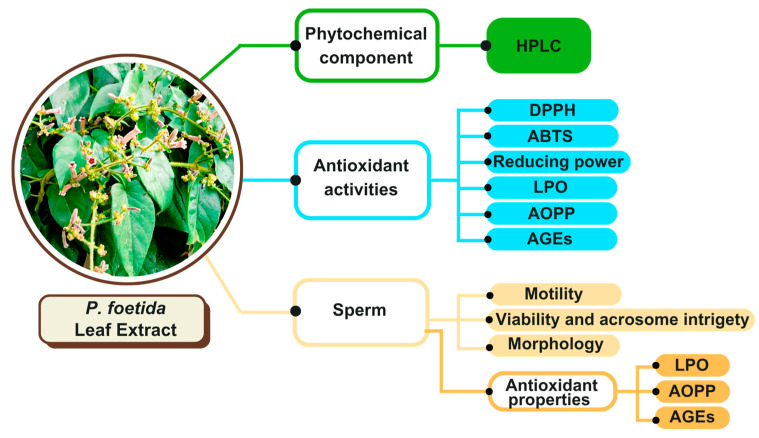
Overview of the experimental research outline. *P. foetida* leaf extract was analyzed for phytochemical content (HPLC) and antioxidant activity (DPPH, ABTS, reducing power, LPO, AOPP, and AGEs). Extracts were applied to frozen–thawed sperm (0.1375–2.20 mg/mL), and sperm quality and antioxidant properties in the supernatant (LPO, AOPP, and AGEs) were assessed.

The collection of mature *P. foetida* leaves took place in October 2023 in the Doi Saket district of Chiang Mai, Thailand. The authentication of the samples (voucher number 0023380) was performed at the Herbarium, Faculty of Pharmacy at Chiang Mai University. The leaves were cleaned and dried at 60 °C. After drying, they were ground into a fine powder and kept at 4 °C until further use. The leaf powder was extracted using 80 °C distilled water for 3 min [47]. Finally, with a ratio of *P. foetida* leaf powder and distilled water of 1:10 *w*/*v*, the solutions were filtered through tea filter paper and diluted using distilled water in preparation for the experiment. The plant extract was dried and lyophilized with 12.5% yield. The overall research activities are shown in a graphical diagram (Figure 1).

### 2.2. Experimental Design

Semen samples from Charolais cattle were purchased from Numchuea Wongwi Company Ltd., Bangkok, Thailand. All protocols in this study were approved by the Ethics Committee of the Faculty of Medicine, Chiang Mai University (26/2024). The semen samples were kept in liquid nitrogen at −196 °C before use. After thawing, the samples were centrifuged at 1500 rpm for 5 min, and the sperm pellets were subsequently washed twice with Krebs solution (pH 7.4). The sperm concentration was diluted to 10 × 10^6^ sperm/mL, and 500 µL of the sample was pipetted into each test tube. The six groups were subsequently prepared as outlined: G1 = control (Krebs solution); G2 = *P. foetida* 0.1375 mg/mL; G3 = *P. foetida* 0.275 mg/mL; G4 = *P. foetida* 0.55 mg/mL; G5 = *P. foetida* 1.10 mg/mL; and G6 = *P. foetida* 2.20 mg/mL. First, 500 µL of each of these groups was put into its respective test tube containing a semen sample. The test tubes were then mixed and incubated at 37 °C. Each treatment group was assessed for sperm motility, sperm morphology, and sperm viability at 2 h post incubation. Each solution was subjected to centrifugation at 2500 rpm for 5 min to separate the sperm pellets from the supernatant. The sperm pellets were subjected to two washes with phosphate-buffered saline (PBS), which had a pH of 7.4. Additionally, the supernatant and sperm pellets were preserved at −20 °C for later antioxidant analysis.

### 2.3. Analysis of Phytochemical Content by High-Performance Liquid Chromatography (HPLC)

High-performance liquid chromatography (HPLC) equipped with a diode array detector (Shimadzu SIL-20AC Prominence Autosampler, Tokyo, Japan) [15,48] was used to analyze the phytochemical composition of *P. foetida*. Chromatographic separation was performed on a Purospher^®^ Star RP-18 (Agilent 1260 Infinity Binary LC, Santa Clara, CA, USA) endcapped column (150 × 4.60 mm, 5 µm particle size). The mobile phase was composed of 92% solvent A (0.1% formic acid in water) and 8% solvent B (acetonitrile), and this composition was held constant during the first 10 min. Subsequently, the proportion of solvent B was increased to 14% at 24 min, 23% at 35 min, and 24% at 60 min. A sample volume of 10 µL was injected for analysis. The wavelengths used for detection were 250, 330, and 360 nm, with spectral data collected in the range of 200–400 nm [22,49]. Phytochemical compound identification relied on the comparison of retention times and UV–vis spectral characteristics of the separated peaks to those of established reference standards.

### 2.4. Antioxidant Characteristics

#### 2.4.1. The Inhibition of 2,2-Diphenyl-1-Picrylhydrazyl (DPPH) Radical Determination

The leaf extract of *P. foetida* was evaluated for its free radical scavenging activity by using the DPPH assay [15]. First, 50 µL of the leaf extract solutions at various concentrations were added to 150 µL of 0.004% DPPH methanolic solution. The mixtures were incubated in the dark for 30 min before measuring absorbance at 515 nm with a microplate reader (BioTek Synergy H1 Hybrid Microplate Reader, BioTek Instrument, Winooski, VT, USA). The positive control in the experiment was gallic acid; the results were expressed as a percentage of inhibition and calculated as follows:

$$\;\%\;\mathrm{inhibition}\;=\frac{A_{\left(\mathrm{DPPH}\right)}-A_{\left(P.\;\mathrm{foetida}\right)}}{A_{\left(\mathrm{DPPH}\right)}}\times 100$$

#### 2.4.2. The Inhibition of 2,2′-Aziobis-[3-Ethylbenzthiazoline-6-Sulfonic Acid] (ABTS) Determination

The leaf extract of *P. foetida* was evaluated for its free radical scavenging activity by ABTS radical cation decolorization assay [15]. The preparation of the ABTS solution involved combining stock solution of ABTS (7 mM) with potassium persulfate solution (2.4 mM). After storing the prepared mixture at 4 °C in darkness for 16 h, the stock solution was diluted using distilled water to obtain an absorbance measured 0.7 at 734 nm. Subsequently, the mixing of 200 µL of the ABTS working solution with 50 µL of leaf extract at different concentrations was incubated in the dark for 30 min. Absorbance readings were obtained at 734 nm using a microplate reader (BioTek Synergy H1 Hybrid Microplate Reader, BioTek Instrument, Winooski, VT, USA). The positive control in the experiment was gallic acid; the results were expressed as a percentage of inhibition and calculated as follows:

$$\%\;\mathrm{inhibition}\;=\frac{A_{\left(\mathrm{ABTS}\right)}-A_{\left(P.\;\mathrm{foetida}\right)}}{A_{\left(\mathrm{ABTS}\right)}}\times 100$$

#### 2.4.3. Reducing Power Determination

The Fe (III)-Fe (II) reducing assay was used to measure the reducing power of *P. foetida* [15]. Initially, each test tube containing 20 µL of leaf extract at different concentrations received 250 µL of PBS (0.2 M; pH: 6.6) and 250 µL of a 1% potassium ferricyanide (K_3_Fe(CN)_6_) solution. The solutions were incubated at 50 °C for 20 min. Then, 250 µL of 10% trichloroacetic acid (TCA) was added, and the mixture underwent centrifugation (1000 rpm for 10 min). Subsequently, 250 µL of the supernatant was collected and then mixed with 250 µL of distilled water and then with 500 µL of 1% ferric chloride solution. Absorbance readings were obtained at 700 nm using a microplate reader (BioTek Synergy H1 Hybrid Microplate Reader, BioTek Instrument, Winooski, VT, USA). The positive control in the experiment was gallic acid.

### 2.5. The Inhibition of Lipid Peroxidation Determination

The thiobarbituric acid-reactive species (TBARS) assay was conducted to determine the ability of *P. foetida* leaf extract to inhibit lipid peroxidation [15]. Initially, 125 µL of linoleic acid emulsion (2.14 M in PBS 0.2 M; pH: 7.4), 12.5 µL of 0.07 M ferrous sulfate (FeSO_4_) solution, and 50 µL of leaf extract at various concentrations were added to test tubes. Subsequently, 100 µL of distilled water was added to each solution, and the samples were incubated at room temperature for 30 min. Then, 225 µL of 85% sodium chloride (NaCl), 500 µL of 10% TCA, and 100 µL of thiobarbituric acid (TBA) were transferred into the test tubes, and the mixtures and the mixtures were subsequently boiled for 30 min at 95 °C. Finally, after cooling, the mixtures were analyzed at a wavelength of 532 nm using a microplate reader (BioTek Synergy H1 Hybrid Microplate Reader, BioTek Instrument, Winooski, VT, USA). The positive control in the experiment was gallic acid; the results were expressed as a percentage of inhibition and calculated as follows:

$$\%\;\mathrm{inhibition}\;=\frac{{\mathrm{OD}}_{\left(\mathrm{control}\right)}-{\mathrm{OD}}_{\left(\mathrm{sample}\right)}}{{\mathrm{OD}}_{\left(\mathrm{control}\right)}}\times 100$$

### 2.6. Inhibition of Advanced Oxidation Protein Products (AOPP) Formation

The inhibition of AOPP activity by *P. foetida* leaf extract was determined using a previously reported method [15]. Initially, each test tube of 50 µL of *P. foetida* leaf extract at different concentrations was combined with 135 µL of a bovine serum albumin (BSA) solution (1 mg/mL in PBS 0.2 M; pH: 7.4) and 15 µL of 0.07 M ferrous sulfate (FeSO_4_) solution. After mixing, the solution was incubated in the dark for 30 min. Subsequently, 50 µL of 1.16 M potassium iodide (KI) solution was added, followed by an additional 2 min of incubation. Finally, 20 µL of absolute acetic acid was added to the solution. Then, 200 µL of the supernatant was placed in a 96-well plate, and the absorbance was recorded at 340 nm using a microplate reader (BioTek Synergy H1 Hybrid Microplate Reader, BioTek Instrument, Winooski, VT, USA). The results were expressed as a percentage of inhibition and calculated as follows:

$$\;\%\;\mathrm{inhibition}\;=\frac{{\mathrm{OD}}_{\left(\mathrm{control}\right)}-{\mathrm{OD}}_{\left(\mathrm{sample}\right)}}{{\mathrm{OD}}_{\left(\mathrm{control}\right)}}\times 100$$

### 2.7. Inhibition of Advanced Glycation End Product (AGE) Formation

The inhibition of AGEs by *P. foetida* leaf extract was determined according to a previously reported method [15]. Initially, 50 µL of *P. foetida* leaf extract with different concentrations was combined with 50 µL of BSA (1 mg/mL in 0.2 M PBS; pH 7.4), and 50 µL of 1 M D-glucose solution was mixed and incubated at 37 °C for 24 h. After incubation, the solutions were measured for fluorescence intensity at an excitation wavelength of 360 nm and an emission wavelength of 460 nm using a microplate reader (BioTek Synergy H1 Hybrid Microplate Reader, BioTek Instrument, Winooski, VT, USA). The results were expressed as a percentage of inhibition and calculated as follows:

$$\;\%\;\mathrm{inhibition}\;=\frac{{\mathrm{OD}}_{\left(\mathrm{control}\right)}-{\mathrm{OD}}_{\left(\mathrm{sample}\right)}}{{\mathrm{OD}}_{\left(\mathrm{control}\right)}}\times 100$$

### 2.8. Sperm Quality Tests

#### 2.8.1. Sperm Motility

Sperm motility was assessed according to the criteria established in a previous study [14] and in line with WHO guidelines [2,50]. The motility types evaluated included progressive motility (spermatozoa displaying active, linear movement, regardless of speed), non-progressive motility (spermatozoa lacking linear movement), circular motility (spermatozoa moving in a circular pattern), and immobility (spermatozoa showing no movement or considered dead). From each of the six groups, 20 µL of sperm solution was transferred to a Neubauer hemocytometer chamber (Boeco, Hamburg, Germany) for analysis. At least 200 sperm cells were counted at 400× magnification using a light microscope (Olympus CH-BI45-2, Olympus, Tokyo, Japan) and categorized for each test tube.

#### 2.8.2. Viability and Acrosome Integrity of Sperm Measurement

A viability test was conducted to distinguish dead spermatozoa from immotile and live ones. Initially, 20 µL of the sperm solution and 20 µL of trypan blue were mixed together. Subsequently, 10 µL of the mixture was placed onto a glass slide and evenly smeared [14]. The slides from all samples were then allowed to air-dry prior to applying Giemsa stain. In the final step, 200 spermatozoa per sample were counted and evaluated under a light microscope at 1000× magnification. The results consisted of the number of viable (unstained) and non-viable (stained) spermatozoa, along with the status of acrosome integrity, categorized as intact or detached.

#### 2.8.3. Sperm Morphology

Sperm morphology was evaluated from smears prepared on glass slides and stained with both trypan blue and Giemsa stain. The stained smears were examined under a bright-field microscope at 1000× magnification using oil immersion. Classification of spermatozoa was conducted based on a previous study [14,18,51,52,53,54,55,56] and the standard criteria established by the WHO [2,50]. Accordingly, each sperm defect was further categorized into four morphological patterns: normal, abnormal head, abnormal head and tail, and abnormal tail [14]. Two hundred spermatozoa were evaluated per sample.

### 2.9. Antioxidant Properties of Sperm Assay

#### 2.9.1. Lipid Peroxidation (LPO) Assay

The analysis of 100 µL sperm supernatant in Krebs solution was carried out using the TBARS method described by Laoung-on et al. [14,15]. Upon completion of the assay, the absorbance of the supernatant was measured using a microplate reader at 532 nm (BioTek Synergy H1 Hybrid Microplate Reader, BioTek Instruments, Winooski, VT, USA).

#### 2.9.2. Inhibition of Formation of Advanced Oxidation Protein Products (AOPPs)

The assay was conducted using a 100 µL sample of sperm supernatant in Krebs solution and following the AOPP procedure outlined by Laoung-on et al. [14,15]. The absorbance of the supernatant was measured using a microplate reader at 340 nm (BioTek Synergy H1 Hybrid Microplate Reader, BioTek Instruments, Winooski, VT, USA).

#### 2.9.3. Suppression of Advanced Glycation End Products (AGEs) Formation

A 100 µL volume of supernatant was dispensed into each well of a 96-well plate, and AGEs were detected using a BioTek Synergy H1 microplate reader (BioTek Instruments, Winooski, VT, USA) with wavelengths of excitation at 360 nm and emission at 460 nm [14,15].

### 2.10. Statistical Analysis

The results were demonstrated as mean ± SD, and data normality was evaluated using the Kolmogorov–Smirnov test. Statistical analysis of mean values for DPPH, ABTS, reducing power, LPO, AOPPs, AGEs, sperm quality, and total oxidative status in sperm was performed using one-way ANOVA for statistical analysis, followed by Tukey’s test to compare multiple groups. For parameters with non-normal distribution, the Kruskal–Wallis test was applied, followed by the Mann–Whitney U-test to detect statistical differences between groups. All experiments were carried out in triplicate, and statistical significance was defined as *p* < 0.05.

## 3. Results

### 3.1. High-Performance Liquid Chromatography (HPLC) Analysis

**Figure 2:**
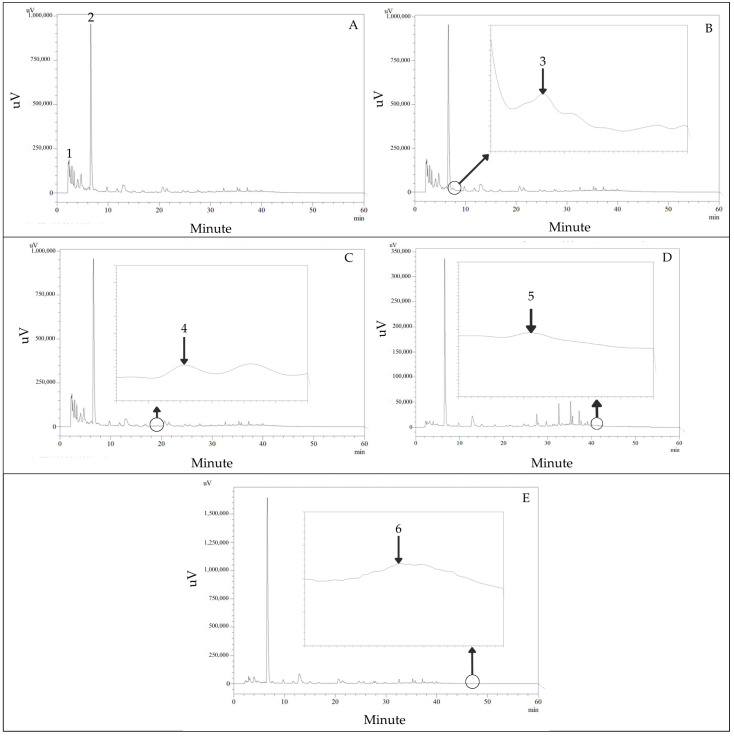
HPLC chromatograms of phenolic compounds are shown in *P. foetida* (**A**–**C**) at a detection wavelength of 280 nm. Flavonoids presented in *P. foetida* (**D**,**E**) at detection wavelengths 330 and 360 nm. Peak identification: peak 1, gallic acid; peak 2, chlorogenic acid; peak 3, caffeic acid; peak 4, ferulic acid; peak 5, quercetin; peak 6, kaempferol.

The HPLC screening for phenolic compounds (280 nm) and flavonoids (330 and 360 nm) was sufficiently separated within 60 min in the *P. foetida* leaf extract. The phenolic compounds are shown in Figure 2A–C. The flavonoids are shown in Figure 2D,E. The retention time and standard UV spectrum of the phytochemical standard were used for identification. The gallic acid, chlorogenic acid, caffeic acid, ferulic acid, quercetin, and kaempferol were found in *P. foetida* leaf extract, and the retention times were 2.58, 6.82, 7.64, 18.08, 40.69, and 47.70 min, respectively. The amounts of the same compounds were 0.43, 1.94, 0.07, 0.03, 0.20, and 0.03 µg/mg plant extract, respectively.

### 3.2. Antioxidant Properties

**Figure 3:**
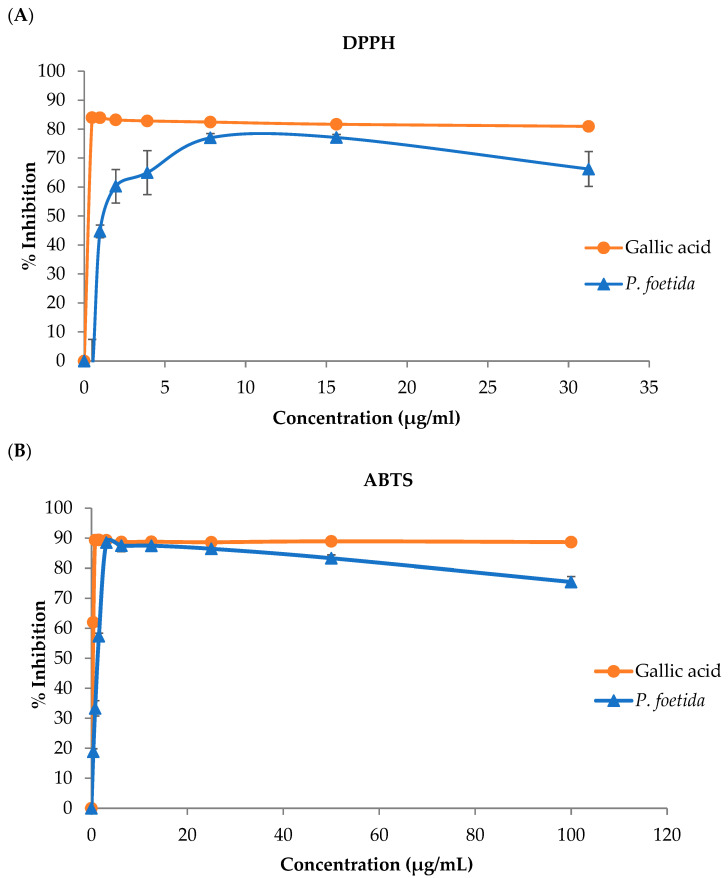

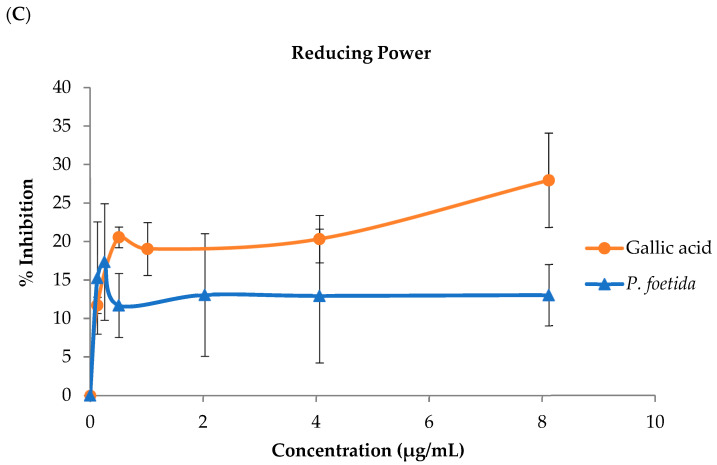
The percentage of inhibition of DPPH (**A**), ABTS (**B**), and reducing power (**C**) of *P. foetida* leaf aqueous extract. Data are represented as mean values ± SD.

The 2,2-diphenyl-1-Picrylhydrazyl (DPPH); 2,2′-azino-di-[3-Ethylbenzthiazoline sulfonate] (ABTS); and reducing Fe (III) to Fe (II) scavenging assays were used to investigate the antioxidant potential of *P. foetida* leaf extract. The half-maximal inhibitory concentration (IC50) for DPPH radical scavenging and the ABTS radical scavenging of the *P. foetida* were presented (Table 1). The leaf extracts exhibited lower radical scavenging activities against DPPH and ABTS radicals as well as lower Fe (III) to Fe (II) radical scavenging compared to gallic acid used as the standard, as shown in Figure 3A–C, respectively.

### 3.3. Lipid Peroxidation (LPO), Advanced Oxidation Protein Products (AOPPs), and Advanced Glycation End Products (AGEs)

**Figure 4:**
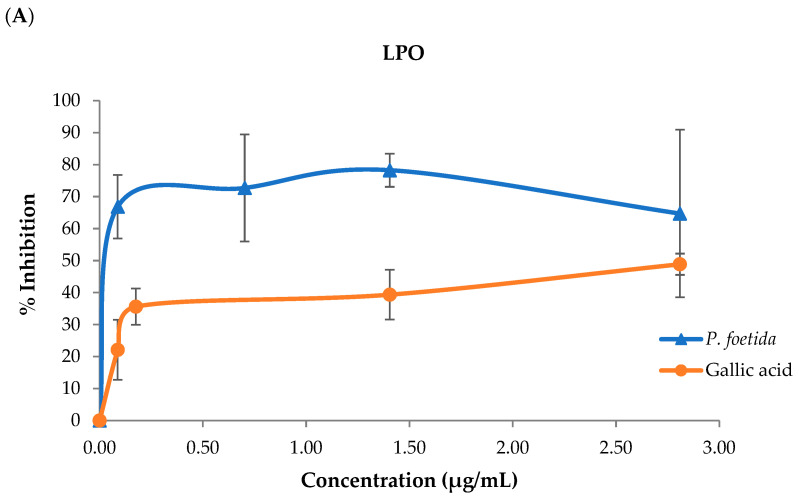

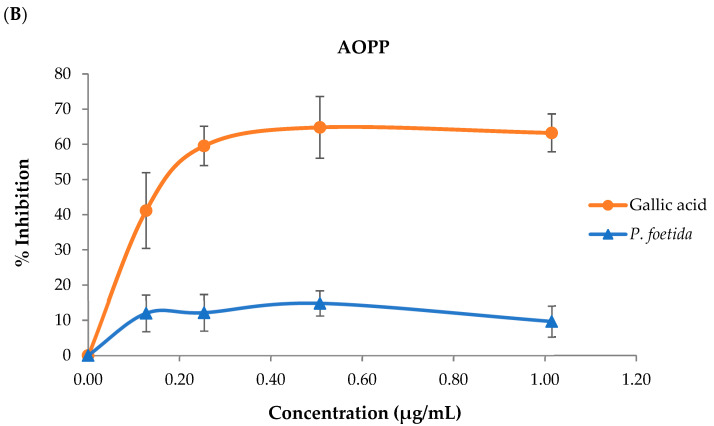
The percentage of inhibition of LPO (**A**) and AOPP (**B**) of *P. foetida* leaf aqueous extract. Data are represented as mean ± SD.

The percentage of LPO inhibition in the *P. foetida* leaf extract is shown in Figure 4A, which indicates that the leaf extract inhibited the LPO formation. The *P. foetida* leaf extract has more potential to inhibit LPO formation than gallic acid. Furthermore, the percentage of *P. foetida* leaf extract inhibition of AOPP formation is shown in Figure 4B, which demonstrates that the *P. foetida* leaf extract has lower potential to inhibit AOPP formation than gallic acid. However, AGEs could not be detected in this leaf extract.

### 3.4. Sperm Quality Test

#### 3.4.1. The Effect of *P. foetida* on Sperm Motility

Charolais cattle sperm samples were combined with the following solutions: G1 = Krebs solution (control); G2 = *P. foetida* 0.1375 mg/mL; G3 = *P. foetida* 0.275 mg/mL; G4 = *P. foetida* 0.55 mg/mL; G5 = *P. foetida* 1.10 mg/mL; and G6 = *P. foetida* 2.20 mg/mL (Table 2). The progressive motility of all treatment groups was significantly decreased when compared to the control group. The number of immotile sperm was significantly increased when compared with the control group.

#### 3.4.2. The Effect of *P. foetida* on Sperm Viability and Acrosome Integrity

The results for sperm viability and acrosome integrity are presented in Table 3. There were no significant differences in the numbers of intact and detached acrosomes of viable and dead sperm observed among cattle sperm treated with *P. foetida* extract at all concentrations when compared to the control group.

#### 3.4.3. The Effect of *P. foetida* on Sperm Morphology

**Figure 5:**
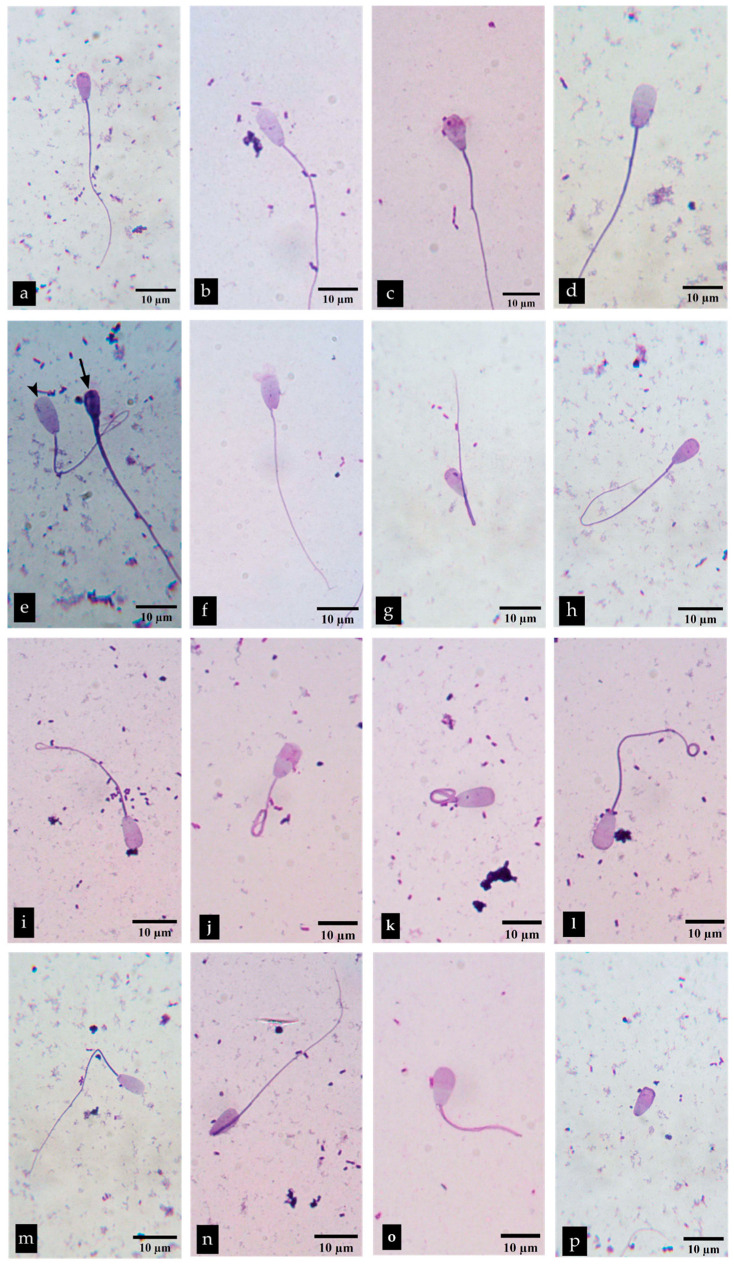
Bright-field images of normal and abnormal morphology of Charolais cattle sperm stained with trypan blue and Giemsa after incubation with *P. foetida* for 2 h: normal sperm (**a**); abnormal head or tail (**b**–**p**); tapered head (**b**); pyriform head (**c**); macrocephalus head (**d**); ⮟ macrocephalus and ⬇ microcephalus head (**e**); abaxial (**f**); distal midpiece reflex (DMR) (**g**); hairpin tail (**h**); tail fold (**i**); coil tail (**j**); dag tail (**k**); tail tip coil (**l**); bent tail (**m**); bent neck (**n**); short tail (**o**); detached head (**p**). Showed at 1000× magnification.

The results for sperm morphology are demonstrated in Table 4 and Figure 5. A significant decrease in the number of normal sperm was observed in the groups treated with 1.10 and 2.20 mg/mL *P. foetida* compared to the control group. However, the 1.10 mg/mL group showed no significant difference from the groups treated with 0.1375, 0.275, and 0.55 mg/mL. A significant decrease in the number of sperm with abnormal heads was observed at concentrations of 0.1375, 0.55, and 1.10 mg/mL compared with the control group. Both head and tail abnormalities were significantly increased in the 1.10 and 2.20 mg/mL groups compared to the control. Moreover, tail abnormalities in all of the concentrations showed no statistically significant difference when compared to the control group, except the 0.1375 mg/mL group. Briefly, a statistically significant increase in abnormal sperm count compared to normal sperm was observed at 1.10 and 2.20 mg/mL.

### 3.5. Antioxidant Characteristics in Sperm

#### 3.5.1. The Inhibition of Lipid Peroxidation (LPO)

The most effective antioxidant characteristics in a cell-free system were observed in *P. foetida*, which was subsequently used to evaluate its antioxidant activity in sperm.

**Figure 6:**
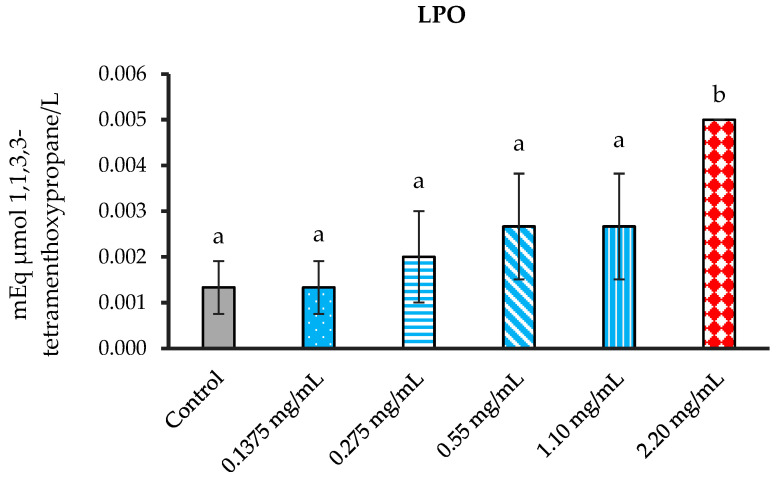
The mean of LPO formation in cattle sperm was evaluated for the control and samples treated with *P. foetida* at concentrations of 0.1375, 0.275, 0.55, 1.10, and 2.20 mg/mL. ^a, b^ Different letters above the bars indicate statistically significant differences between groups, while the same letters indicate no significant difference. The data are shown as mean ± SD.

Each group of sperm solution, which was treated with various doses of leaf extract, was analyzed for LPO and additional antioxidant statuses. The *P. foetida* concentrations were designed to test for capacity of scavenged free radicals. Each group of supernatants was measured for LPO levels. The result demonstrated no significant difference in all treatment groups when compared with the control group. However, the supernatant in the concentration of 2.20 mg/mL significantly differed from the control and the other groups (Figure 6).

#### 3.5.2. Suppression of Advanced Oxidation Protein Products (AOPPs) Formation

Each group of sperm suspension, which was treated with various concentrations of leaf extract, was analyzed for AOPPs and additional antioxidant statuses. The *P. foetida* concentrations were designed to test for capacity of scavenged free radicals. Each group of supernatants was measured for AOPP levels.

**Figure 7:**
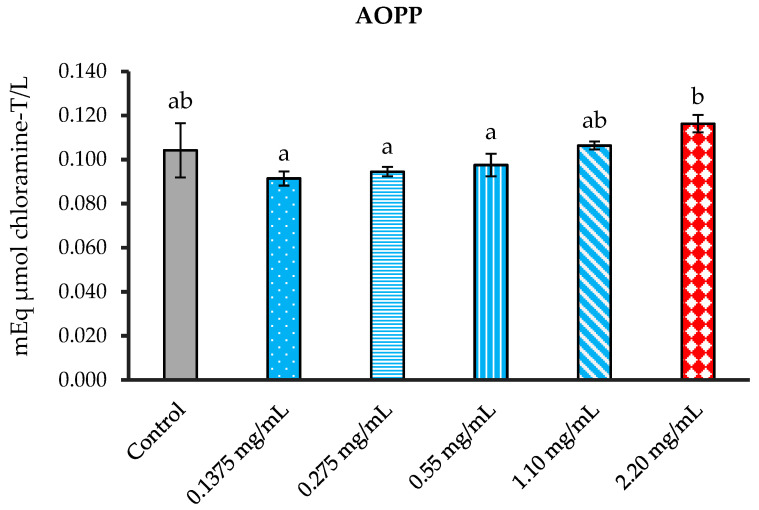
The mean of AOPP formation in cattle sperm was evaluated for the control and samples treated with *P. foetida* at concentrations of 0.1375, 0.275, 0.55, 1.10, and 2.20 mg/mL. ^a, b^ Different letters above the bars indicate statistically significant differences between groups, while the same letters indicate no significant difference. The data are shown as mean ± SD.

The results showed no statistically significant differences in AOPP levels between the control group and all treatment concentrations. In contrast, the 2.20 mg/mL concentration showed a significant difference compared to the 0.1375, 0.275, and 0.55 mg/mL concentrations (Figure 7).

#### 3.5.3. Inhibition of Formation of Advanced Glycation End Products (AGEs)

The *P. foetida* extract was specifically selected to test its potential free radical scavenging activity. The supernatant from each group was measured for AGE levels.

**Figure 8:**
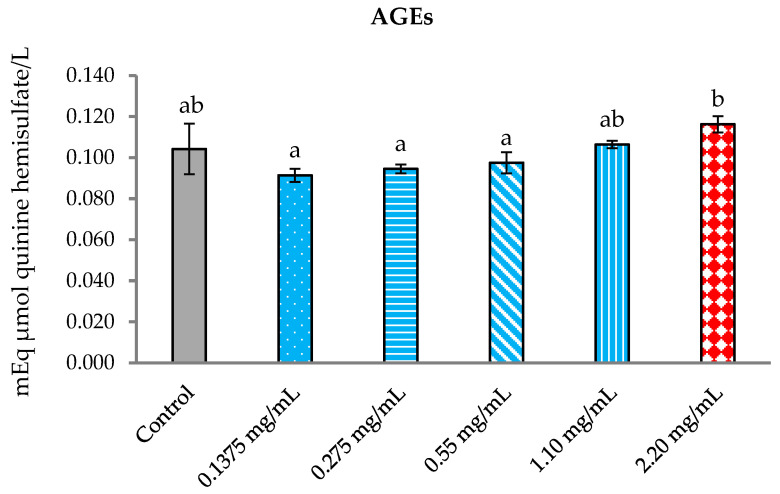
The mean of AGE formation in cattle sperm was evaluated for the control and samples treated with *P. foetida* at concentrations of 0.1375, 0.275, 0.55, 1.10, and 2.20 mg/mL. ^a, b^ Different letters above the bars indicate statistically significant differences between groups, while the same letters indicate no significant difference. The data are shown as mean ± SD.

The results demonstrated no statistically significant difference between the control group and the other treatment groups. However, the 2.20 mg/mL treatment group exhibited a statistically significant difference when compared to 0.1375, 0.275, and 0.55 mg/mL concentrations (Figure 8).

## 4. Discussion

In the present phytochemical investigation of *P. foetida*, dried leaves were extracted using distilled water as the solvent to simulate typical herbal tea preparation practices. Our preliminary screening using the colorimetric assay, a qualitative testing method, indicated the presence of terpenoids, cardiac glycosides, flavonoids, tannins, and phenols [47]. In the next analysis, we used HPLC to determine the types and quantities of phytochemicals in this plant extract. HPLC revealed key phenolic and flavonoid compounds, including gallic acid, chlorogenic acid, caffeic acid, ferulic acid, quercetin, and kaempferol. In this study, the extraction method employed was decoction, a traditional technique that utilizes heat and boiling water to extract phytochemical compounds from plants, which was carried out at 80 °C for 3–5 min. This process simulates a traditional herbal tea preparation method that is simple, convenient, and suitable for daily consumption. However, these compounds of *P. foetida* found by HPLC technique were in low amounts of phenols, which differs from *M. oleifera* leaf tea that was found to have high quantities of these compounds when extracted by the same method [22]. Recently, a study on *Bombax ceiba* (*B. ceiba*) stamen reported low phenolic content, similar to this study, but higher phenolic content when extracted by ultrasonic extraction, which can preserve and enhance these compounds [23]. In the analysis of flavonoid content in the present study, it was found that the flavonoid compound exhibited a similar trend to that of phenolics. An interesting observation from the study on *B. ceiba* stamen extract is that a comparison of extraction methods revealed that ultrasonic-assisted extraction had a lower flavonoid content than decoction, which is opposite to the results for the phenolic content [23]. This report reflects that different extraction methods can affect the concentration of each compound in plants, and even if one extraction method concentrates a high amount of a particular compound, it does not necessarily result in similarly high levels of other compounds [23]. These findings are supported by the other existing literature, which has indicated that extraction parameters such as temperature, solvent type, extraction duration, and extraction method significantly affect the type and concentration of bioactive compounds in plant materials, thereby impacting the biological activity of the resulting extracts [23,27,57,58,59].

The ABTS assay revealed higher antioxidant activity of *P. foetida* leaf extract than the DPPH assay. This observation was further confirmed by the lower IC50 value obtained from the ABTS assay, indicating that the ABTS method enhanced radical scavenging capability. These results are consistent with the research on aqueous lotus petal extract in assessing antioxidant activity using the same methods [15]. The variation in antioxidant activity is likely attributable to the different interactions between solvent polarity and the phytochemical constituents of the extract [57,58]. It has been reported that the polarity of the extraction solvent, the concentration of bioactive compounds, and the specific antioxidant assay employed are positively correlated variables that influence the outcome, and the present findings are consistent with these data [59,60,61,62]. These factors may help explain the differences in antioxidant activity measurements reported in our study.

The results of the Fe^3+^ to Fe^2+^ reducing power assay in this study were lower than fifty percent inhibition; therefore, the IC50 value could not be calculated. Although phenolic compounds capable of donating electrons were present, their low levels may be the cause of the limited reducing power. This outcome corresponds with the earlier literature, which reported that the presence of multiple antioxidant agents in plant or herbal extracts may result in decreased reducing power due to antagonistic interactions among active constituents [63]. To assess the antioxidative capacity of *P. foetida* against lipid degradation, an in vitro lipid peroxidation model was constructed using linoleic acid, which was induced by FeSO_4_. The extract demonstrated significant inhibition of lipid oxidation, likely attributable to the presence of phenolic compounds, particularly chlorogenic acid (CGA), which was found at higher concentrations compared to other constituents. CGA has been reported to inhibit lipase and lipoxygenase, which are enzymes involved in promoting lipid oxidation [64]. In contrast, when tested in a protein oxidation model using D-glucose-induced bovine serum albumin (BSA), *P. foetida* extract exhibited relatively low antioxidant activity against advanced oxidation protein products (AOPP), especially in comparison with gallic acid. Previous studies have shown that CGA metabolites such as ferulic acid and caffeic acid contribute significantly to the reduction in AOPP levels [57]. However, the previously noted low concentrations of these metabolites in the aqueous extract of *P. foetida* may underlie its limited efficacy in suppressing AOPP formation.

The present study evaluated the effects of *P. foetida* leaf extract on sperm quality and oxidative stress to determine whether the extract enhances sperm function and quality or induces cellular damage. The results demonstrated that at concentrations below 1.10 mg/mL, the *P. foetida* leaf extract did not significantly alter sperm parameters, including sperm motility, morphology, or acrosomal integrity, when compared to the control. However, at a concentration of 2.2 mg/mL, a significant increase in immotile sperm and abnormalities in the head and tail regions were observed along with a decrease in acrosomal integrity. These findings indicate that high concentrations of the extract may exert cytotoxic effects on spermatozoa. Previous studies have reported that high concentrations of *Azadirachta indica* (neem) extract reduce sperm motility and viability as well as increase the proportion of morphologically abnormal sperm [65]. In addition, the assessment of oxidative stress levels in *P. foetida* leaf extract, as measured by AOPP and AGEs, indicated that all tested concentrations of the extract inhibited oxidative stress, showing no significant differences compared to the normal control group. However, oxidative stress levels in LPO assays at a concentration of 2.2 mg/mL were significantly elevated compared to both the control group and groups treated with lower extract concentrations. Under normal physiological conditions, the cell membrane, which is composed of saturated fatty acids, cholesterol, and glycosphingolipids, is directly exposed to reactive oxygen species (ROS), rendering it highly susceptible to oxidative damage and structural instability [66,67]. Although *P. foetida* leaf extract comprises various phytochemicals with diverse antioxidant activities, it can be postulated that the observed reductions in sperm motility, morphology, viability, and acrosomal integrity following exposure to the extract at a concentration of 2.2 mg/mL may be attributed to excessive generation of reactive oxygen species (ROS) induced by the extract. This elevated ROS production likely initiates lipid peroxidation (LPO), a process in which ROS attacks polyunsaturated fatty acids (PUFAs), key components of the sperm plasma membrane, thereby altering membrane fluidity, compromising membrane integrity, and ultimately impairing sperm function [68]. The previous literature has reported that aldehydes, which are byproducts of the LPO process, can bind to proteins within the electron transport chain (ETC), thereby initiating a self-perpetuating cycle of ROS generation that leads to ongoing oxidative stress, compromising the structural integrity of sperm cells, inducing DNA damage, and ultimately leading to decreased sperm viability and acrosomal integrity [67,68]. Additionally, it is possible that a high concentration of *P. foetida* extract may affect the structure and functional properties of the mitochondrial membrane, leading to a loss of membrane potential, which in turn may impair the energy production system and interfere with components essential for sperm motility, potentially resulting in reduced motility and increased morphological abnormalities [69]. Although in our study, the aqueous extract of *P. foetida* leaves contained phytochemicals possessing potential antioxidant properties, it neither enhanced nor reduced sperm quality in concentrations up to 1.10 mg/mL when compared with the control group. It can be assumed that *P. foetida* can be safely consumed without causing sperm damage in concentrations up to 1.10 mg/mL, while at higher concentrations of 2.2 mg/mL, the *P. foetida* leaf extract may induce structural and functional damage to sperm. This study employed an aqueous extraction method simulating typical tea consumption and was performed solely on sperm cells in vitro, which does not reflect the processes of absorption and metabolic transformation occurring in vivo. Thus, additional studies in animal models and a clinical trial in human should be undertaken to obtain more comprehensive data on the safe consumption of mature *P. foetida* leaf extracts for human health and then apply them to benefit applications.

## 5. Conclusions

The aqueous extract from mature leaves of *P. foetida* was found to contain measurable amounts of phenolic and flavonoid compounds and exhibited antioxidant activity. The extract, at concentrations not more than 1.10 mg/mL, showed no significant difference in effect on sperm quality compared to the control group. However, a concentration of 2.20 mg/mL caused increased damage to sperm quality. Therefore, further in vivo studies are needed to investigate the effects of the extract on various physiological systems. Additionally, alternative extraction techniques should be explored to isolate other bioactive compounds, which may have potential applications in promoting reproductive health and supporting additional physiological functions.

## Figures and Tables

**Table 1:** The half-maximal inhibitory concentration (IC50) values of *P. foetida* leaf extracts with different methods by the DPPH and ABTS scavenging activity.

Samples	IC50 (µg/mL)	
	DPPH	ABTS
Gallic acid	0.29 ± 0.01 ^a^	0.39 ± 0.01 ^a^
*P. foetida*	1.43 ± 0.08 ^b^	1.31 ± 0.03 ^b^

^a, b^ Different letters above the variables indicate significant differences between groups (*p* < 0.05). DPPH and ABTS were evaluated using one-way ANOVA followed by Tukey’s test.

**Table 2:** The number of Charolais cattle sperm motility treated with *P. foetida* and a control group.

Groups	Concentrations (mg/mL)	Number of Motile Sperm			Number of Immotile Sperm
		Progressive	Non-Progressive	Circle	
Control	0 mg/mL	25.00 ± 19.47 ^a^	41.33 ± 7.37 ^ab^	0.33 ± 0.58 ^a^	133.67 ± 12.10 ^a^
*P* *. foetida*	0.1375 mg/mL	0.00 ± 0.00 ^b^	44.00 ± 6.56 ^ab^	0.00 ± 0.00 ^a^	156.00 ± 6.56 ^bc^
	0.275 mg/mL	0.00 ± 0.00 ^b^	53.33 ± 8.02 ^b^	0.00 ± 0.00 ^a^	146.67 ± 8.02 ^ab^
	0.55 mg/mL	0.00 ± 0.00 ^b^	55.33 ± 4.93 ^b^	0.00 ± 0.00 ^a^	144.67 ± 4.93 ^ab^
	1.10 mg/mL	0.00 ± 0.00 ^b^	42.67 ± 1.53 ^ab^	0.00 ± 0.00 ^a^	157.33 ± 1.53 ^bc^
	2.20 mg/mL	0.00 ± 0.00 ^b^	29.33 ± 9.87 ^a^	0.00 ± 0.00 ^a^	170.67 ± 9.87 ^c^

Different superscript letters ^a, b, c^ in the same column indicate statistically significant differences between groups, while the same letters show no significant difference. Statistical significance was evaluated using one-way ANOVA with Tukey’s test (*p* < 0.05). The results are reported as mean ± SD.

**Table 3:** The number of Charolais cattle sperm viability and acrosome integrity treated with *P. foetida* and a control group.

Groups	Concentrations (mg/mL)	Number of Viable Sperm		Number of Dead Sperm	
		Intact	Detached	Intact	Detached
Control	0 mg/mL	65.00 ± 12.77	32.00 ± 16.64	3.33 ± 4.16	100.33 ± 9.07
*P* *. foetida*	0.1375 mg/mL	66.00 ± 4.58	33.67 ± 7.51	2.67 ± 3.06	100.33 ± 12.01
	0.275 mg/mL	61.67 ± 4.04	42.00 ± 9.52	2.33 ± 1.53	96.33 ± 8.50
	0.55 mg/mL	59.67 ± 6.81	38.33 ± 4.04	4.00 ± 3.61	102.00 ± 6.56
	1.10 mg/mL	52.33 ± 11.93	38.00 ± 9.85	3.00 ± 1.00	109.67 ± 9.81
	2.20 mg/mL	48.33 ± 5.69	19.33 ± 10.02	9.67 ± 5.51	122.67 ± 11.50

Statistical analysis of all parameters was performed using one-way ANOVA, followed by Tukey’s test with *p* ≤ 0.05. Data are reported as mean ± SD.

**Table 4:** The number of normal and abnormal Charolais cattle sperm morphology treated with *P. foetida* and a control group.

Groups	Concentrations (mg/mL)	Number of Normal Sperm	Number of Abnormal Sperm		
			Head Only	Head and Tail	Tail Only
Control	0 mg/mL	39.00 ± 16.52 ^a^	51.00 ± 7.21 ^a^	59.33 ± 24.79 ^a^	50.67 ± 10.02 ^ab^
*P. foetida*	0.1375 mg/mL	19.33 ± 14.01 ^ab^	17.00 ± 1.73 ^c^	78.33 ± 26.35 ^ab^	85.33 ± 12.86 ^c^
	0.275 mg/mL	16.00 ± 4.36 ^ab^	29.00 ± 5.00 ^bc^	86.00 ± 5.57 ^abc^	69.00 ± 7.00 ^bc^
	0.55 mg/mL	22.33 ± 2.89 ^ab^	32.67 ± 6.35 ^b^	92.33 ± 4.16 ^abc^	52.67 ± 4.93 ^ab^
	1.10 mg/mL	10.00 ± 4.58 ^b^	30.67 ± 2.08 ^bc^	125.67± 4.04 ^c^	33.67 ± 2.52 ^a^
	2.20 mg/mL	10.33 ± 5.51 ^b^	40.67 ± 7.02 ^ab^	120.67 ± 11.24 ^bc^	28.33 ± 11.93 ^a^

Different superscript letters ^a, b, c^ in the same column indicate statistically significant differences between groups, while the same letters show no significant difference. Statistical significance was evaluated using one-way ANOVA with Tukey’s test (*p* < 0.05). The results are reported as mean ± SD.

## Data Availability

All authors declare that the data supporting the findings of this study are available within the article.
